# Identification of marker genes for spinal cord injury

**DOI:** 10.3389/fmed.2024.1364380

**Published:** 2024-02-23

**Authors:** Zhiwei Luan, Jiayu Zhang, Yansong Wang

**Affiliations:** ^1^Department of Orthopedic Surgery, The First Affiliated Hospital of Harbin Medical University, Harbin, China; ^2^The Key Laboratory of Myocardial Ischemia, Chinese Ministry of Education, Harbin, China; ^3^Department of Hygienic Toxicology, College of Public Health, Harbin Medical University, Harbin, China; ^4^NHC Key Laboratory of Cell Transplantation, Harbin Medical University, Harbin, China; ^5^Heilongjiang Provincial Key Laboratory of Hard Tissue Development and Regeneration, Harbin Medical University, Harbin, China

**Keywords:** cord injury, bioinformatics, WGCNA, LASSO regression, marker genes

## Abstract

**Introduction:**

Spinal cord injury (SCI) is a profoundly disabling and devastating neurological condition, significantly impacting patients’ quality of life. It imposes unbearable psychological and economic pressure on both patients and their families, as well as placing a heavy burden on society.

**Methods:**

In this study, we integrated datasets GSE5296 and GSE47681 as training groups, analyzed gene variances between sham group and SCI group mice, and conducted Gene Ontology (GO) enrichment analysis and Kyoto Encyclopedia of Genes and Genomes (KEGG) enrichment analysis based on the differentially expressed genes. Subsequently, we performed Weighted Gene Correlation Network Analysis (WGCNA) and Lasso regression analyses.

**Results:**

We identified four characteristic disease genes: *Icam1*, *Ch25h*, *Plaur* and *Tm4sf1*. We examined the relationship between SCI and immune cells, and validated the expression of the identified disease-related genes in SCI rats using PCR and immunohistochemistry experiments.

**Discussion:**

In conclusion, we have identified and verified four genes related to SCI: *Icam1*, *Ch25h*, *Plaur* and *Tm4sf1*, which could offer insights for SCI treatment.

## 1 Introduction

Spinal cord injury (SCI) commonly occurs due to various incidents including car accidents, falls, sports-related injuries, earthquakes, among others. The global prevalence of SCI has risen from 236 cases per million people to 1,298 cases. Current estimates place the annual number of SCI cases worldwide between 250,000 and 500,000, with over one million SCI patients in China (1–3). SCI imposes a substantial psychological burden on patients and places heavy strains on families and society. Regeneration and repair of SCI represent some of the most daunting medical challenges, yet effective methods to promote neurological function recovery are lacking. Currently available approaches can only offer supportive relief for individuals who are disabled for life (4–6). Hence, there is an urgent need to develop a safe and efficient treatment for spinal cord injury.

The emergence and advancement of bioinformatics provide new avenues for investigating the pathogenesis and therapeutic targets of SCI. Over the past decade, the continuous progression of gene chips and high-throughput sequencing technology, combined with the extensive use of machine learning and artificial intelligence, have led to the evolution of novel bioinformatics fields, which include biomarker discovery, prediction of drug targets, and research on protein function (7). Whole genome analysis technologies such as high-throughput sequencing and gene chips have been widely adopted globally in the last decade to study gene expression patterns, resulting in the generation of vast amounts of data (8). This has driven the development of data processing and analysis methodology, and has given rise to algorithms or models such as protein–protein interaction (PPI) network, weighted gene correlation network analysis (WGCNA), and receiver operating characteristic curve (ROC), all providing valuable insights for disease mechanism investigation and biomarker discovery (9–11).

In this study, we used WGCNA and LASSO regression analysis to identify four marker genes for SCI, which were subsequently validated through PCR and immunohistochemistry experiments using SCI rats. These findings may offer assistance in future SCI diagnosis and treatment.

## 2 Method

### 2.1 Data download and processing

We utilized the Gene Expression Omnibus (GEO),^1^ a publicly available functional genomics database with diverse gene expression datasets (12). We downloaded datasets GSE5296 and GSE47681 as the training group, and GSE132242 as the validation group. Subsequently, we used Perl software and related code to organize this information into a matrix file comprising sample names and mouse gene names. Following this, we used Perl software and relevant code to segregate mRNA and lncRNA from the gene matrix file, thereby obtaining the lncRNA gene matrix file of SCI. The version of R we use for data analysis is 4.2.0.

### 2.2 Differential genes and functional enrichment

The “Limma” R package (3.52.0) was employed to identify differentially expressed genes (DEGs) in SCI and healthy mice, using a significance threshold of *p* < 0.05. We further utilized the “clusterprofiler” R package (4.4.1) to conduct Gene Ontology (GO) enrichment analysis and Kyoto Encyclopedia of Genes and Genomes (KEGG) enrichment analysis on the selected DEGs (13).

### 2.3 WGCNA and LASSO regression

The R package “WGCNA” (1.72.1) was utilized to perform WGCNA analysis based on different subtypes of CRGs content. Outlier samples were excluded, and the remaining samples were clustered to construct topological overlap matrices and scale-free networks. Utilizing a gene module correlation >0.8 as the filtering criterion, we identified the gene module displaying the strongest clinical correlation. Subsequently, genes were organized within this module to facilitate the subsequent step of core gene screening. An SCI diagnostic model was developed based on LASSO regression coefficients, and the model’s performance was evaluated using ROC curve and area under the curve (AUC). Finally, the model was validated with dataset GSE132242.

### 2.4 Correlation between SCI and immune cells

In order to further investigate the impact of DEG expression on immune cell infiltration, we conducted a single sample gene set enrichment analysis (ssGSEA) using the R language GSVA package (1.44.0). This enabled us to obtain the gene set based on the ssGSEA algorithm for each sample. Combining data analysis of immune cells and immune function, we utilized the R language pheatmap software package to create heatmaps of the dataset, immune cells, and immune function.

### 2.5 Animal experiment

We divided 10 SD rats into two groups equally: the control group and the SCI group. Following anesthesia of the SCI group rats, we removed dorsal hair and made a 1.5 cm incision at the T10 segment of the spinal cord to expose the rat spinal cord. We then used weights dropped from a height to impact the rat spinal cord. One week later, we euthanized the rats and collected their spinal cord tissue for further analysis. This experiment was approved by the Ethics Committee of the First Affiliated Hospital of Harbin Medical University (Ethics approval number: 2023045).

### 2.6 Quantitative PCR assay and immunohistochemistry

To further investigate the variations in risk genes between SCI-affected tissues and normal tissues, we conducted quantitative real-time reverse transcription polymerase chain reaction (RT-PCR) analysis and immunohistochemistry. The experimental protocol for PCR and immunohistochemistry was performed as previously described (14). The relevant primers for PCR are provided in Table 1.

**Table 1:** Primers for qRT-PCR analysis.

Gene	Forward premier	Reverse premier
*Icam1*	TCTTCCTCGGCCTTCCCATA	AGGTACCATGGCCCCAAATG
*Ch25h*	GACCTTCCGTGGTCAACTCA	GGAGATCATGATGCGGTGCT
*Tm4sf1*	TTCCATTCCACAATGTGCTT	GGCCAGTGGAACTACACCTT
*Plaur*	ACAACAACGACACCTTCC	AGTACAGCAGGAGACATCA

Information on antibodies used in immunohistochemistry: Ch25h (Abcam, ab214295), Plaur (Abcam, ab307895), Tm4sf1(Sigma-Aldrich, SAB1307024).

## 3 Result

### 3.1 Differential gene expression and enrichment analysis

**Figure 1:**
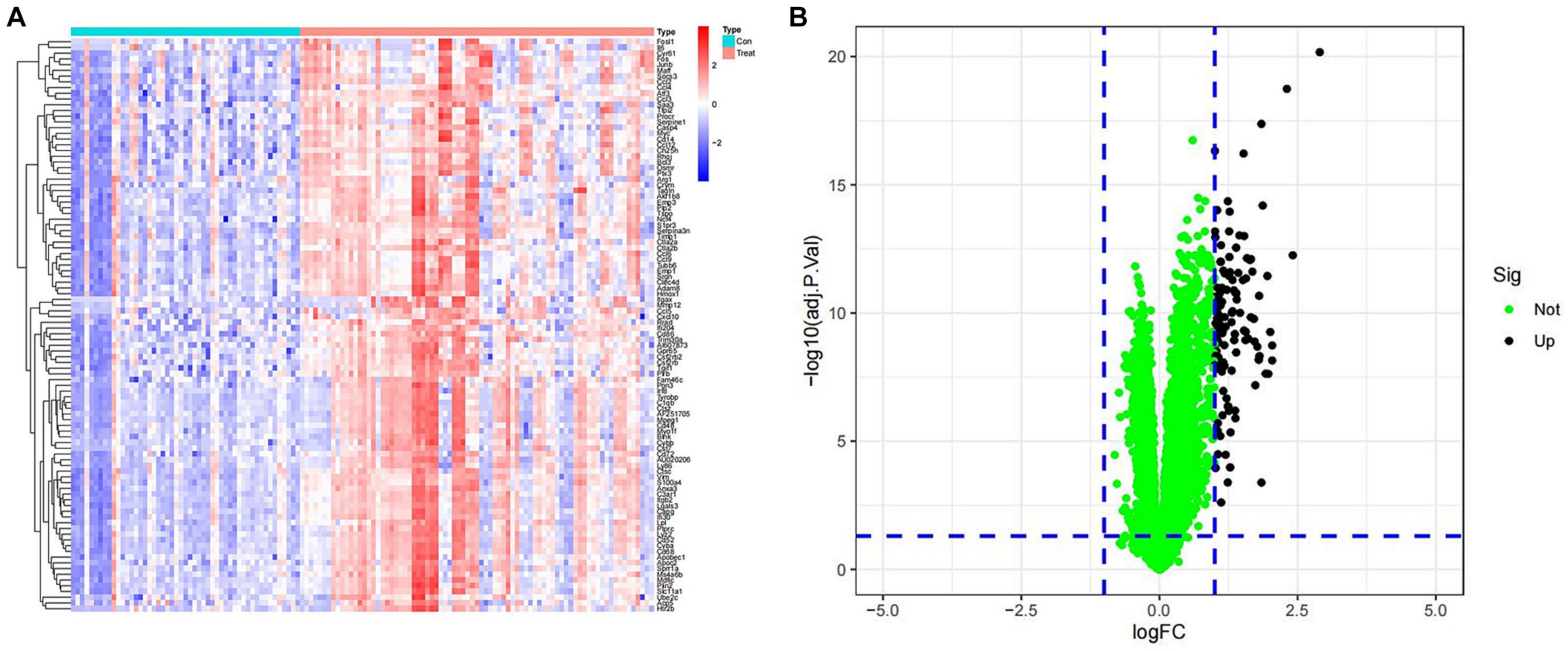
Expression of differentially expressed genes. **(A)** Heat map of DEGs. Blue represents samples from the control group, while red represents samples from the SCI group. The vertical axis is the name of the differentially expressed gene. Red indicates upregulation of gene expression, while blue indicates downregulation of gene expression. **(B)** Volcano map of DEGs. Green dots represent genes with no differences, while black dots represent upregulated differentially expressed genes.

**Figure 2:**
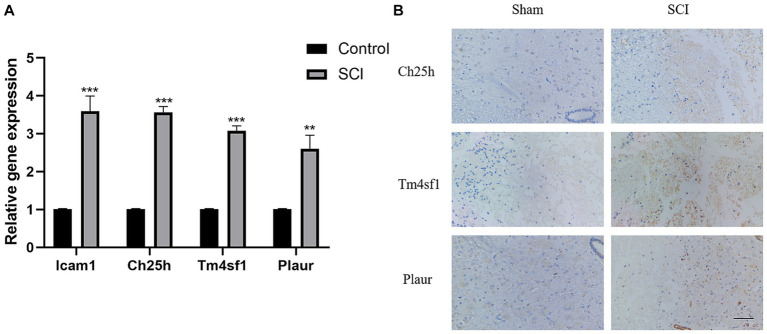
Verification of SCI characteristic genes. **(A)** Verify the expression of Icam1, Ch25h, Plaur and Tm4sf1 in rat tissues using PCR. **(B)** Verify the expression of Ch25h, Plaur and Tm4sf1 in rat tissues using immunohistochemical.

**Figure 3:**
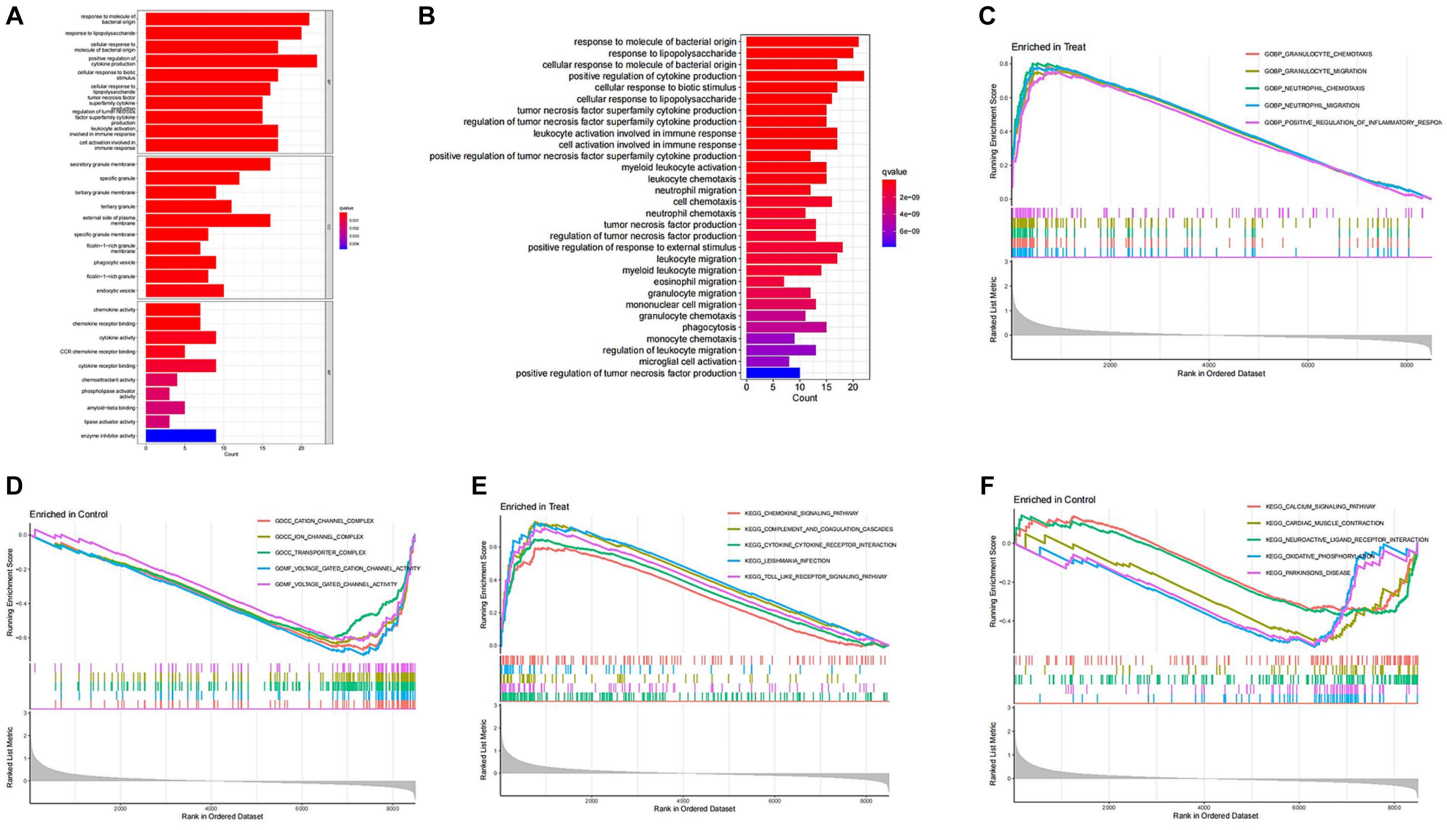
Enrichment analysis of DEGs. **(A,B)** GO enrichment analysis and KEGG enrichment analysis. The vertical axis represents the name of the function/pathway, and the horizontal axis represents the number of genes enriched in each function/pathway. The color of the column represents the significance of enrichment, and the redder the color, the more significant the enrichment of differential genes in that function/pathway. **(C–F)** GSEA enrichment analysis. The horizontal axis represents sorted genes, while the vertical axis represents enriched scores. Different colored curves represent different functions/pathways. The peak of the curve appearing on the upper left side indicates that the function/pathway is active in the SCI group, while the peak of the curve appearing on the lower right side indicates that the function/pathway is active in the control group.

To investigate the differential gene expression between healthy mice and those with SCI, we conducted heat maps and volcano plots of DEGs. Our analysis revealed a significant increase in DEG expression in the SCI group compared to the control group, while no decrease in DEG expression was observed (Figure 1). Subsequently, we performed GO enrichment analysis and KEGG enrichment analysis. The biological processes (BP) involving DEGs were notably enriched in response to molecular stimuli of bacterial origin and response to lipopolysaccharide. Furthermore, the molecular functions (MF) of DEGs were significantly enriched in processes such as secretory granule membrane and specific granule (Figure 2C). The cellular composition (CC) of DEGs was significantly enriched in processes such as chemokine activity and chemokine receptor binding (Figure 3A). KEGG enrichment analysis showed that DEGs are associated with pathways such as myeloid leukemia activation and cell chemotaxis (Figure 3B). Notably, GSEA analysis highlighted differences between the control group and the SCI group, with the former mainly enriched in the calcium signaling pathway and oxidative physiology, while the SCI group exhibited enrichment in neutrophil chemotaxis and neutrophil migration, indicative of the crucial role of immunity in SCI (Figures 3C–F).

### 3.2 Identification of SCI characteristic genes

**Figure 4:**
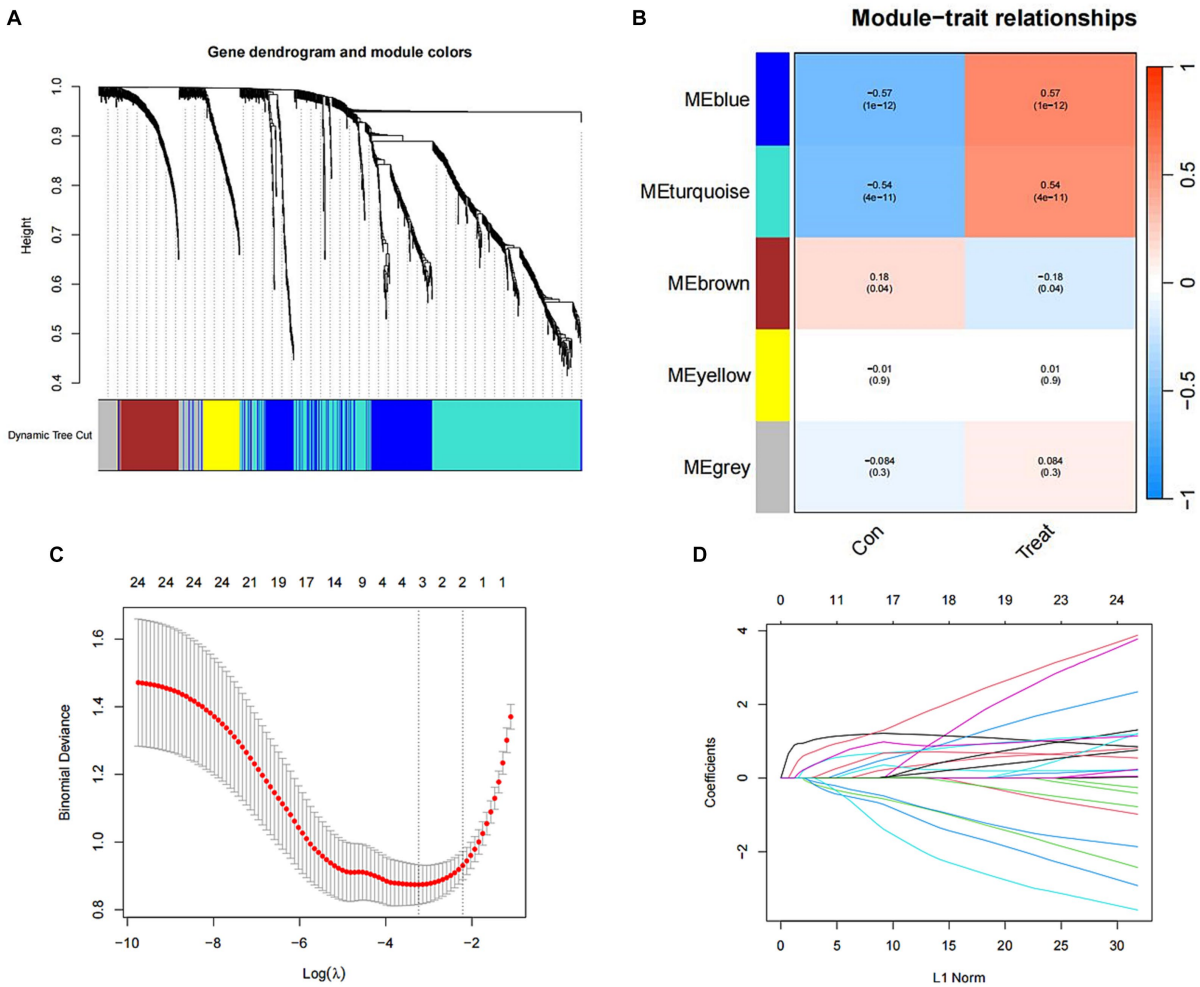
Identification of SCI characteristic genes. **(A)** Gene clustering tree diagram. Cluster genes, where different genes belong to different color modules. **(B)** Heat map of the correlation between modules and clinical characteristics. In each grid, the upper value represents the correlation coefficient, and the lower value represents the *p*-value of the correlation test. The correlation coefficients and *p*-values of different modules screened by WGCNA. The depth of module colors represents the degree of correlation. **(C)** Cross-verified graphics. The abscissa represents the logλ value, and the ordinate represents the error of the cross-validation. The number of points with the smallest cross-validation error corresponds to the number of disease-characteristic genes. **(D)** LASSO regression graph.

Utilizing WGCNA as a method for discovering genes and clinical phenotypes, we explored key genes related to clinical disease phenotypes by integrating DEGs. Following analysis of the positive correlation coefficient, we identified the module with the strongest correlation coefficient. Our results revealed that the blue module [*r* = 0.57, *P* = (1e−12)] displayed the most significant genetic significance. Subsequently, we extracted genes from the blue module, which are considered key genes related to the clinical phenotype (Figures 4A,B). Then LASSO regression analysis was performed to identify 4 characteristic genes associated with SCI: *Icam1*, *Ch25h*, *Plaur* and *Tm4sf1* (Figures 4C,D).

### 3.3 Expression of SCI characteristic genes in different groups

**Figure 5:**
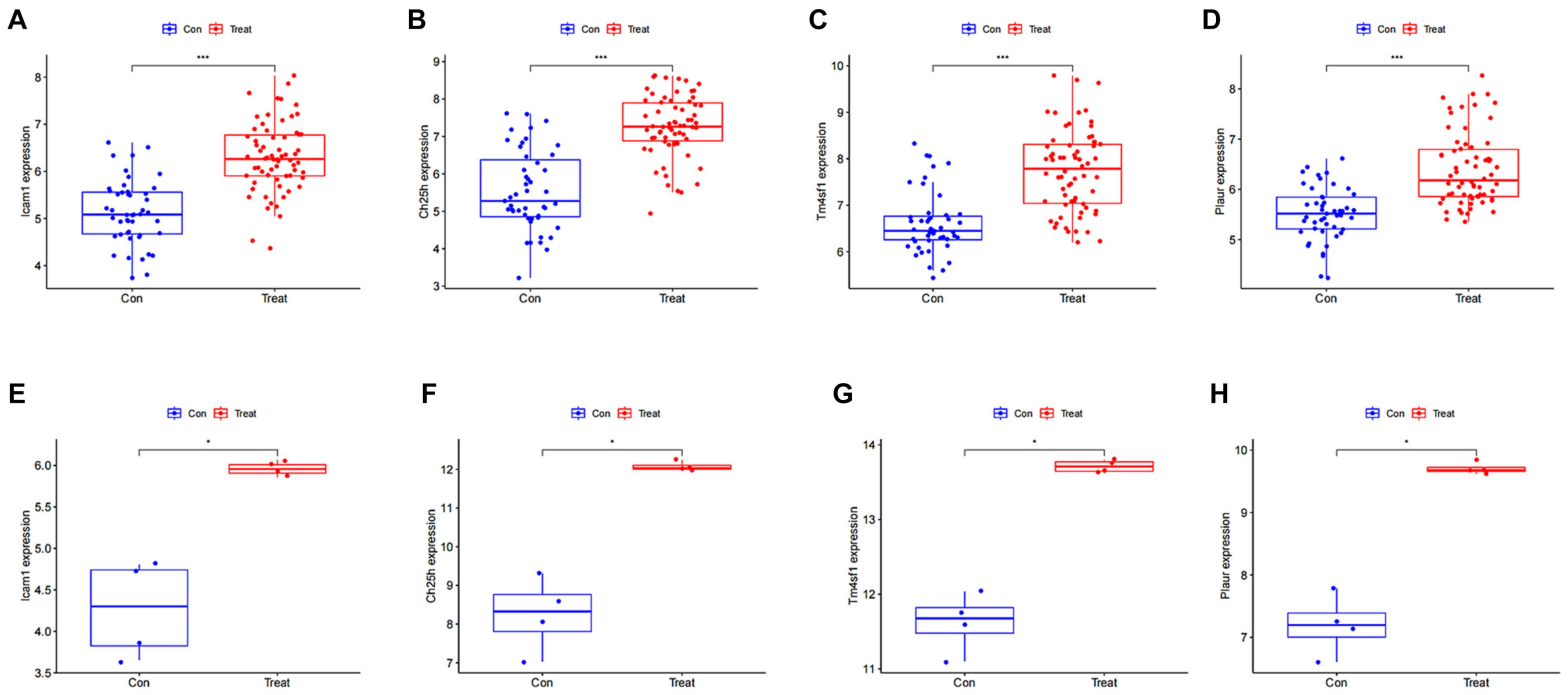
Expression of SCI characteristic genes in different groups. **(A–D)** Differential boxplot of training group. The horizontal axis represents the type of sample, which is divided into control group samples and SCI group samples. The control group is represented in blue, while the SCI group is represented in red. The vertical axis represents the expression level of characteristic genes. **(E–H)** Differential boxplot of the validation group. The difference in the characteristic genes of this disease between the control group and the SCI group (*p*-value <0.05). And the box plot of the SCI group is higher than that of the control group, indicating that the gene is upregulated in the SCI group.

We then analyzed the expression of the four disease characteristic genes in both the control group and the SCI group of mice. Our findings revealed upregulation of *Icam1*, *Ch25h*, *Plaur* and *Tm4sf1* in the SCI group (Figures 5A–D). Moreover, we used dataset GSE132242 as the validation group for analysis, confirming increased expression of these four genes in the SCI group (Figures 5E–H).

### 3.4 ROC curve of SCI characteristic genes

**Figure 6:**
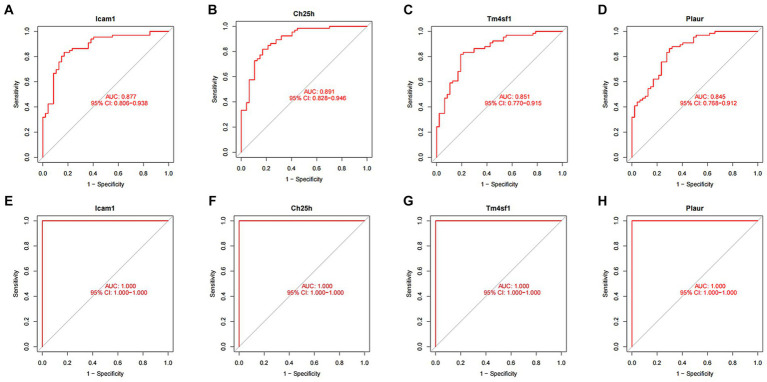
ROC curve of SCI characteristic genes. **(A–D)** ROC curves of four characteristic genes in the training group. The horizontal axis represents the false positive rate, and the vertical axis represents the true positive rate. The larger the area under the curve, the higher the accuracy of the gene as a disease diagnostic gene. **(E)** ROC curves of Icam1, **(F)** Ch25h, **(G)** Tm4sf1, and **(H)** Plaur in the validation group.

We conducted ROC curve analysis to validate the SCI characteristic genes. Our findings revealed AUC values of 0.877, 0.891, 0.851 and 0.845 for the four genes in the training group, indicating relatively high accuracy of the disease characteristic genes we screened (Figures 6A–D). Subsequently, we utilized the validation group to develop ROC curves, further confirming the accuracy of our model (Figures 6E–H).

### 3.5 Immunological correlation analysis of SCI characteristic genes

**Figure 7:**
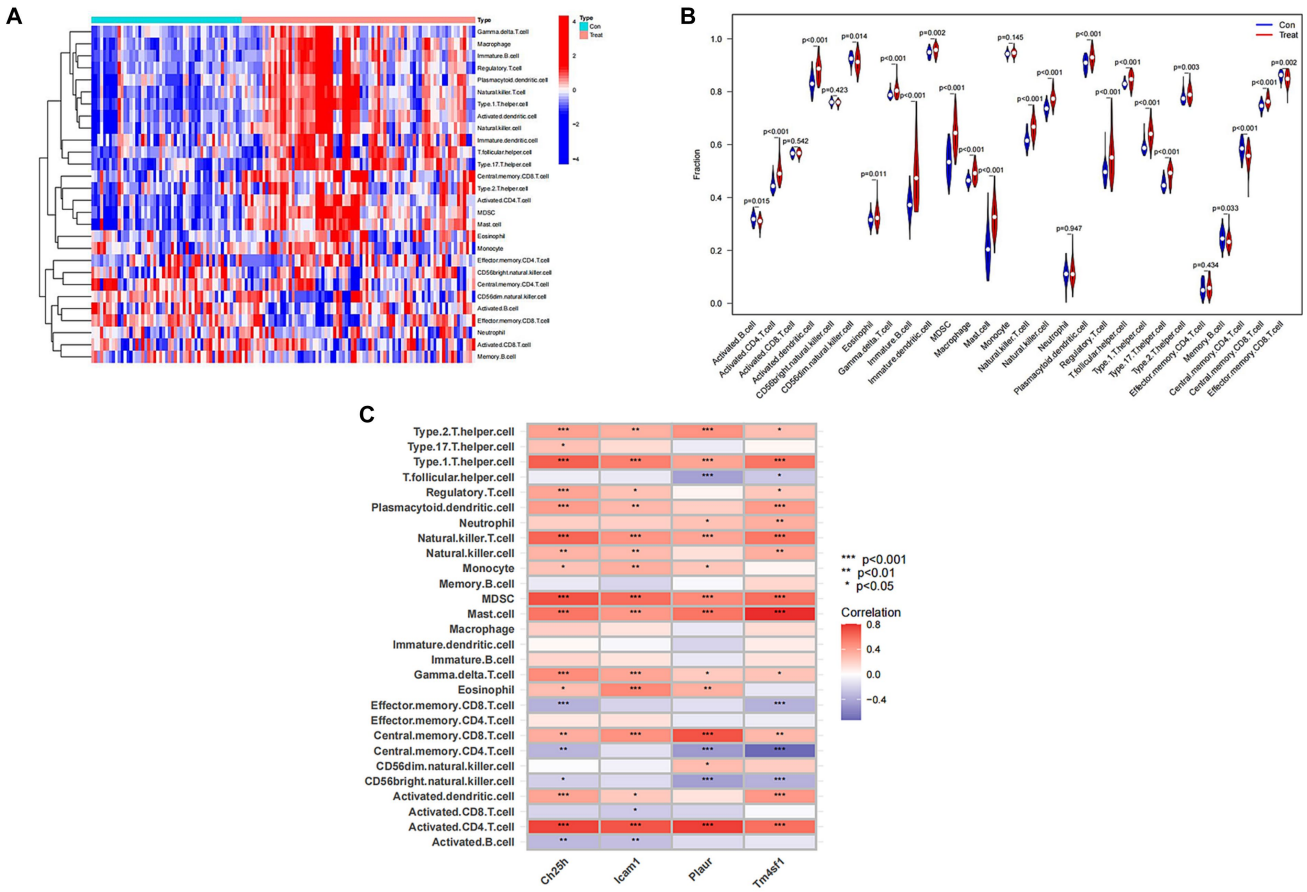
Immunological correlation analysis of SCI characteristic genes. **(A)** Heat map of immune cells. Green represents the control group sample, and red represents the SCI group sample. The vertical axis represents various immune cells. Blue represents low expression, while red represents high expression. **(B)** Violin diagram of immune cell differences. The horizontal axis represents the name of immune cells, and the vertical axis represents the content of immune cells. If *p* < 0.05, it indicates that there is a difference in the immune cells between the control group and the SCI group. **(C)** Correlation analysis between SCI characteristic genes and immune cells. The vertical axis represents the name of immune cells, and the horizontal axis represents the characteristic genes of the disease.

Recognizing the vital role of immunity in SCI progression, we analyzed differences in immune cells between the SCI group and the control group. Our analysis revealed higher expression of most immune cells in the SCI group compared to the control group, with only a few immune cells showing higher expression in the Control group than in the SCI group, such as activated B cells and effector memory CD8 T cells (Figures 7A,B). Additionally, several immune cells are associated with SCI characteristic genes, including regulatory T cells, natural killer cells, and mast cells (Figure 7C).

### 3.6 Validation of SCI characteristic genes

Finally, we validated the previously screened genes through LASSO regression using PCR and immunohistochemistry experiments. We observed higher expression of *Icam1*, *Ch25h*, *Plaur* and *Tm4sf1* in the SCI group rats compared to the control group (Figures 2A,B).

## 4 Discussion

In this study, we initially compared gene expression between mice in the SCI group and those in the control group by utilizing gene heatmaps and volcano plots. Subsequently, we performed GO enrichment analysis and KEGG enrichment analysis on the differentially expressed genes. We identified four characteristic SCI genes through WGCNA and LASSO regression, and verified the model’s accuracy using ROC curves. Furthermore, we found that multiple immune cells are associated with these characteristic genes, implying their potential role in immune regulation. Finally, we validated the characteristic SCI genes through PCR and immunohistochemical experiments.

SCI stands as one of the most debilitating and detrimental central nervous system afflictions, capable of causing spinal cord vascular rupture, rapid neural cell demise, and impaired movement, sensation, and autonomic nervous system function below the affected segment (4, 15). Over the past 30 years, the global incidence rate has surged from 236 cases per million people to 1,298 cases, escalating annually (16, 17). SCI can be categorized into non-traumatic and traumatic SCI based on its causes. Non-traumatic SCI primarily arises from acute and chronic conditions like tumors and intervertebral disc herniation (18, 19), while traumatic SCI is predominantly triggered by traffic accidents, sports-related falls, or violent acts, leading to spinal fractures, dislocations, and subsequent cord compression or rupture (20, 21). SCI profoundly impacts patients’ quality of life, mental and physical well-being, and imposes significant economic burdens on families and society. Consequently, addressing SCI holds substantial societal importance.

Icam-1, a cell membrane surface glycoprotein weighing 90 kDa, was initially found to interact with leukocyte function-associated antigen-1, facilitating the adhesion of white blood cells or tumor cells to endothelial cells. This interaction contributes to inflammatory immune responses or tumor metastasis (22–24). Additionally, Icam-1 can exist in the extracellular matrix as a soluble factor, regulating inflammation and immunity within the body. Exogenous Icam-1 may originate from tumor cell membrane detachment or be secreted into the extracellular matrix in vesicle form (25, 26). Notably, the association between Icam-1 and gastric cancer metastasis has been confirmed through numerous clinical and pathological tissue studies (27).

Ch25h, an interferon-stimulated gene, encodes a product that catalyzes cholesterol oxidation, producing soluble 25-hydroxycholesterol, thereby playing a crucial regulatory role in cholesterol metabolism homeostasis (28). Apart from its pivotal function in cellular metabolism, Ch25h’s role in the body’s immunity has gained increasing attention in recent years (29, 30). Research indicates that TLR ligands and interferon stimulation can induce high Ch25h expression in macrophages and dendritic cells. Ch25h can inhibit IgA generation in B lymphocytes and affect intracellular bacteria growth (31). As an immune transmitter, Ch25h can induce inflammatory factor expression; thus, dysregulated Ch25h can lead to immune pathological changes in the body (32).

Plaur is a multifunctional receptor on the cell surface that can be expressed on various immune active cells such as neutrophils and activated T lymphocytes. In addition, it can also be expressed on endothelial cells and some tumor cells (33). There have been studies on the relationship between Plaur and lung injury in respiratory diseases, and some observations suggest that injury may be related to immune mechanisms (34). Plaur plays an important role in the fibrinolytic system and is also related to the progression of tumors. It can promote the proliferation of tumor cells and facilitate their migration between different regions (35).

The protein encoded by the Tm4sf1 gene is a member of the transmembrane 4 superfamily, mediating signal transduction events regulating cell development, activation, growth, and movement (36). Tm4sf1 is highly expressed in human pancreatic cancer, breast cancer, lung cancer, and other tumors, closely linked to tumor cell growth, migration, and invasion (37–40). It has also been reported to have high expression in human tumor endothelial cells. Reducing Tm4sf1 levels can inhibit cell migration, block cell division, promote cell aging, and inhibit VEGF-A-induced vascular maturation (41). Our data indicates a significant increase in the expression of Icam1, Ch25h, Plaur and Tm4sf1 in the rat SCI group.

In this study, we used data set GSE132242 as the verification group to verify that the expressions of Icam1, Ch25h, Plaur and Tm4sf1 in the SCI group were higher than those in the Control group. This is the first time that dataset GSE132242 has appeared in a related article in bioinformatics analysis. We found four SCI characteristic genes. As a characteristic gene of SCI, Plaur has not been thoroughly studied. We believe that Plaur may be helpful for the identification and treatment of SCI.

In summary, we identified four pathogenic genes related to SCI using the WGCNA model and LASSO regression, validating them through PCR and immunohistochemical. Our findings may offer new insights into SCI-related diseases.

## Data availability statement

The datasets presented in this study can be found in online repositories. The names of the repository/repositories and accession number(s) can be found in the article/Supplementary material.

## Ethics statement

The animal study was approved by the Ethics Committee of the First Affiliated Hospital of Harbin Medical University (Ethics approval number: 2023045). The study was conducted in accordance with the local legislation and institutional requirements.

## Author contributions

ZL: Data curation, Investigation, Software, Writing – original draft. JZ: Formal analysis, Project administration, Validation, Writing – original draft. YW: Funding acquisition, Resources, Writing – review & editing.

## Footnotes

^1^ http://www.ncbi.nlm.nih.gov/geo
