# Supplementary material for: Identification of marker genes for spinal cord injury
Source: Front Med (Lausanne). 2024 Feb 23;11:1364380. doi: 10.3389/fmed.2024.1364380 (PMC10921937; doi:10.3389/fmed.2024.1364380)
Supplement: Supplementary file 1 [file Table_1.DOCX]

**Figure1A**

library(limma)

library(pheatmap)

inputFile="merge.txt"

logFCfilter=1

adj.P.Val.Filter=0.05

setwd("C:\\Users\\lenovo\\Desktop\\146wgcna\\06.diff")

rt=read.table(inputFile, header=T, sep="\t", check.names=F)

rt=as.matrix(rt)

rownames(rt)=rt[,1]

exp=rt[,2:ncol(rt)]

dimnames=list(rownames(exp),colnames(exp))

data=matrix(as.numeric(as.matrix(exp)),nrow=nrow(exp),dimnames=dimnames)

data=avereps(data)

data=data[rowMeans(data)>0,]

sampleName1=c()

files=dir()

files=grep("s1.txt$", files, value=T)

for(file in files){

rt=read.table(file, header=F, sep="\t", check.names=F)

geneNames=as.vector(rt[,1])

uniqGene=unique(geneNames)

sampleName1=c(sampleName1, uniqGene)

}

sampleName2=c()

files=dir()

files=grep("s2.txt$", files, value=T)

for(file in files){

rt=read.table(file, header=F, sep="\t", check.names=F)

geneNames=as.vector(rt[,1])

uniqGene=unique(geneNames)

sampleName2=c(sampleName2, uniqGene)

}

conData=data[,sampleName1]

treatData=data[,sampleName2]

data=cbind(conData,treatData)

conNum=ncol(conData)

treatNum=ncol(treatData)

Type=c(rep("con",conNum),rep("treat",treatNum))

design <- model.matrix(~0+factor(Type))

colnames(design) <- c("con","treat")

fit <- lmFit(data,design)

cont.matrix<-makeContrasts(treat-con,levels=design)

fit2 <- contrasts.fit(fit, cont.matrix)

fit2 <- eBayes(fit2)

allDiff=topTable(fit2,adjust='fdr',number=200000)

allDiffOut=rbind(id=colnames(allDiff),allDiff)

write.table(allDiffOut, file="all.txt", sep="\t", quote=F, col.names=F)

outData=rbind(id=paste0(colnames(data),"_",Type),data)

write.table(outData, file="normalize.txt", sep="\t", quote=F, col.names=F)

diffSig=allDiff[with(allDiff, (abs(logFC)>logFCfilter & adj.P.Val < adj.P.Val.Filter )), ]

diffSigOut=rbind(id=colnames(diffSig),diffSig)

write.table(diffSigOut, file="diff.txt", sep="\t", quote=F, col.names=F)

diffGeneExp=data[row.names(diffSig),]

diffGeneExpOut=rbind(id=paste0(colnames(diffGeneExp),"_",Type), diffGeneExp)

write.table(diffGeneExpOut, file="diffGeneExp.txt", sep="\t", quote=F, col.names=F)

geneNum=50

diffSig=diffSig[order(as.numeric(as.vector(diffSig$logFC))),]

diffGeneName=as.vector(rownames(diffSig))

diffLength=length(diffGeneName)

hmGene=c()

if(diffLength>(2*geneNum)){

hmGene=diffGeneName[c(1:geneNum,(diffLength-geneNum+1):diffLength)]

}else{

hmGene=diffGeneName

}

hmExp=data[hmGene,]

Type=c(rep("Con",conNum),rep("Treat",treatNum))

names(Type)=colnames(data)

Type=as.data.frame(Type)

pdf(file="heatmap.pdf", width=10, height=8)

pheatmap(hmExp,

annotation=Type,

color = colorRampPalette(c("blue", "white", "red"))(50),

cluster_cols =F,

show_colnames = F,

scale="row",

fontsize = 8,

fontsize_row=7,

fontsize_col=8)

dev.off()

**Figure1B**

library(ggplot2)

logFCfilter=1

adj.P.Val.Filter=0.05

inputFile="all.txt"

setwd("C:\\Users\\lenovo\\Desktop\\146wgcna\\07.volcano")

rt=read.table(inputFile, header=T, sep="\t", check.names=F)

Sig=ifelse((rt$adj.P.Val<adj.P.Val.Filter) & (abs(rt$logFC)>logFCfilter), ifelse(rt$logFC>logFCfilter,"Up","Down"), "Not")

rt=cbind(rt, Sig=Sig)

p=ggplot(rt, aes(logFC, -log10(adj.P.Val)))+

geom_point(aes(col=Sig))+

scale_color_manual(values=c("green", "black", "red"))+

xlim(-5,5)+

labs(title = " ")+

geom_vline(xintercept=c(-logFCfilter,logFCfilter), col="blue", cex=1, linetype=2)+

geom_hline(yintercept= -log10(adj.P.Val.Filter), col="blue", cex=1, linetype=2)+

theme(plot.title=element_text(size=16, hjust=0.5, face="bold"))

p=p+theme_bw()

pdf(file="volcano.pdf", width=6, height=5.1)

print(p)

dev.off()

**Figure2A**

library(clusterProfiler)

library(org.Hs.eg.db)

library(enrichplot)

library(ggplot2)

library(circlize)

library(RColorBrewer)

library(dplyr)

library(ComplexHeatmap)

pvalueFilter=0.05

qvalueFilter=0.05

colorSel="qvalue"

if(qvalueFilter>0.05){

colorSel="pvalue"

}

ontology.col=c("#00AFBB", "#E7B800", "#90EE90")

setwd("C:\\Users\\lenovo\\Desktop\\146wgcna\\08.GO")

rt=read.table("diff.txt", header=T, sep="\t", check.names=F)

genes=unique(as.vector(rt[,1]))

entrezIDs=mget(genes, org.Hs.egSYMBOL2EG, ifnotfound=NA)

entrezIDs=as.character(entrezIDs)

gene=entrezIDs[entrezIDs!="NA"]

#gene=gsub("c\\(\"(\\d+)\".*", "\\1", gene)

kk=enrichGO(gene=gene, OrgDb=org.Hs.eg.db, pvalueCutoff=1, qvalueCutoff=1, ont="all", readable=T)

GO=as.data.frame(kk)

GO=GO[(GO$pvalue<pvalueFilter & GO$qvalue<qvalueFilter),]

write.table(GO, file="GO.txt", sep="\t", quote=F, row.names = F)

showNum=10

if(nrow(GO)<30){

showNum=nrow(GO)

}

pdf(file="barplot.pdf", width=10, height=15)

bar=barplot(kk, drop=TRUE, showCategory=showNum, label_format=30, split="ONTOLOGY", color=colorSel) + facet_grid(ONTOLOGY~., scale='free')

print(bar)

dev.off()

pdf(file="bubble.pdf", width=10, height=15)

bub=dotplot(kk, showCategory=showNum, orderBy="GeneRatio", label_format=30, split="ONTOLOGY", color=colorSel) + facet_grid(ONTOLOGY~., scale='free')

print(bub)

dev.off()

**Figure2B**

library(clusterProfiler)

library(org.Hs.eg.db)

library(enrichplot)

library(ggplot2)

library(circlize)

library(RColorBrewer)

library(dplyr)

library(ComplexHeatmap)

pvalueFilter=0.05

qvalueFilter=0.05

colorSel="qvalue"

if(qvalueFilter>0.05){

colorSel="pvalue"

}

setwd("C:\\biowolf\\wgcnaDiagnostic\\09.KEGG")

rt=read.table("diff.txt", header=T, sep="\t", check.names=F)

genes=unique(as.vector(rt[,1]))

entrezIDs=mget(genes, org.Hs.egSYMBOL2EG, ifnotfound=NA)

entrezIDs=as.character(entrezIDs)

rt=data.frame(genes, entrezID=entrezIDs)

gene=entrezIDs[entrezIDs!="NA"]

#gene=gsub("c\\(\"(\\d+)\".*", "\\1", gene)

kk <- enrichKEGG(gene=gene, organism="hsa", pvalueCutoff=1, qvalueCutoff=1)

KEGG=as.data.frame(kk)

KEGG$geneID=as.character(sapply(KEGG$geneID,function(x)paste(rt$genes[match(strsplit(x,"/")[[1]],as.character(rt$entrezID))],collapse="/")))

KEGG=KEGG[(KEGG$pvalue<pvalueFilter & KEGG$qvalue<qvalueFilter),]

write.table(KEGG, file="KEGG.txt", sep="\t", quote=F, row.names = F)

showNum=30

if(nrow(KEGG)<showNum){

showNum=nrow(KEGG)

}

pdf(file="barplot.pdf", width=9, height=7)

barplot(kk, drop=TRUE, showCategory=showNum, label_format=130, color=colorSel)

dev.off()

pdf(file="bubble.pdf", width = 9, height = 7)

dotplot(kk, showCategory=showNum, orderBy="GeneRatio", label_format=130, color=colorSel)

dev.off()

**Figure2C-F**

library(limma)

library(org.Hs.eg.db)

library(clusterProfiler)

library(enrichplot)

inputFile="all.txt"

gmtFile="c2.cp.kegg.Hs.symbols.gmt"

setwd("C:\\Users\\lexb\\Desktop\\geoARG\\11.GSEA")

rt=read.table(inputFile, header=T, sep="\t", check.names=F)

logFC=as.vector(rt[,2])

names(logFC)=as.vector(rt[,1])

logFC=sort(logFC, decreasing=T)

gmt=read.gmt(gmtFile)

kk=GSEA(logFC, TERM2GENE=gmt, pvalueCutoff = 1)

kkTab=as.data.frame(kk)

kkTab=kkTab[kkTab$p.adjust<0.05,]

write.table(kkTab,file="GSEA.result.txt",sep="\t",quote=F,row.names = F)

termNum=5

kkUp=kkTab[kkTab$NES>0,]

if(nrow(kkUp)>=termNum){

showTerm=row.names(kkUp)[1:termNum]

gseaplot=gseaplot2(kk, showTerm, base_size=8, title="Enriched in Treat")

pdf(file="GSEA.treat.pdf", width=6.5, height=5)

print(gseaplot)

dev.off()

}

termNum=5

kkDown=kkTab[kkTab$NES<0,]

if(nrow(kkDown)>=termNum){

showTerm=row.names(kkDown)[1:termNum]

gseaplot=gseaplot2(kk, showTerm, base_size=8, title="Enriched in Control")

pdf(file="GSEA.con.pdf", width=6.5, height=5)

print(gseaplot)

dev.off()

}

**Figure3A-B**

library(limma)

library(WGCNA)

expFile="normalize.txt"

setwd("C:\\biowolf\\wgcnaDiagnostic\\11.WGCNA")

rt=read.table(expFile, header=T, sep="\t", check.names=F)

rt=as.matrix(rt)

rownames(rt)=rt[,1]

exp=rt[,2:ncol(rt)]

dimnames=list(rownames(exp),colnames(exp))

data=matrix(as.numeric(as.matrix(exp)),nrow=nrow(exp),dimnames=dimnames)

data=avereps(data)

data=data[apply(data,1,sd)>0.7,]

Type=gsub("(.*)\\_(.*)", "\\2", colnames(data))

conCount=length(Type[Type=="con"])

treatCount=length(Type[Type=="treat"])

datExpr0=t(data)

gsg = goodSamplesGenes(datExpr0, verbose = 3)

if (!gsg$allOK)

{

# Optionally, print the gene and sample names that were removed:

if (sum(!gsg$goodGenes)>0)

printFlush(paste("Removing genes:", paste(names(datExpr0)[!gsg$goodGenes], collapse = ", ")))

if (sum(!gsg$goodSamples)>0)

printFlush(paste("Removing samples:", paste(rownames(datExpr0)[!gsg$goodSamples], collapse = ", ")))

# Remove the offending genes and samples from the data:

datExpr0 = datExpr0[gsg$goodSamples, gsg$goodGenes]

}

sampleTree = hclust(dist(datExpr0), method = "average")

pdf(file = "1_sample_cluster.pdf", width = 12, height = 9)

par(cex = 0.6)

par(mar = c(0,4,2,0))

plot(sampleTree, main = "Sample clustering to detect outliers", sub="", xlab="", cex.lab = 1.5, cex.axis = 1.5, cex.main = 2)

abline(h = 20000, col = "red")

dev.off()

clust = cutreeStatic(sampleTree, cutHeight = 20000, minSize = 10)

table(clust)

keepSamples = (clust==1)

datExpr0 = datExpr0[keepSamples, ]

traitData=data.frame(Con=c(rep(1,conCount),rep(0,treatCount)),

Treat=c(rep(0,conCount),rep(1,treatCount)))

row.names(traitData)=colnames(data)

fpkmSamples = rownames(datExpr0)

traitSamples =rownames(traitData)

sameSample=intersect(fpkmSamples,traitSamples)

datExpr0=datExpr0[sameSample,]

datTraits=traitData[sameSample,]

sampleTree2 = hclust(dist(datExpr0), method = "average")

traitColors = numbers2colors(datTraits, signed = FALSE)

pdf(file="2_sample_heatmap.pdf",width=12,height=12)

plotDendroAndColors(sampleTree2, traitColors,

groupLabels = names(datTraits),

main = "Sample dendrogram and trait heatmap")

dev.off()

enableWGCNAThreads()

powers = c(1:20)

sft = pickSoftThreshold(datExpr0, powerVector = powers, verbose = 5)

pdf(file="3_scale_independence.pdf",width=9,height=5)

par(mfrow = c(1,2))

cex1 = 0.9

plot(sft$fitIndices[,1], -sign(sft$fitIndices[,3])*sft$fitIndices[,2],

xlab="Soft Threshold (power)",ylab="Scale Free Topology Model Fit,signed R^2",type="n",

main = paste("Scale independence"));

text(sft$fitIndices[,1], -sign(sft$fitIndices[,3])*sft$fitIndices[,2],

labels=powers,cex=cex1,col="red");

abline(h=0.90,col="red")

plot(sft$fitIndices[,1], sft$fitIndices[,5],

xlab="Soft Threshold (power)",ylab="Mean Connectivity", type="n",

main = paste("Mean connectivity"))

text(sft$fitIndices[,1], sft$fitIndices[,5], labels=powers, cex=cex1,col="red")

dev.off()

sft

softPower =sft$powerEstimate

adjacency = adjacency(datExpr0, power = softPower)

softPower

pdf(file="3_softConnectivity.pdf", width=9, height=5)

k <- softConnectivity(datE=datExpr0,power=softPower)

#sizeGrWindow(10, 5)

par(mfrow=c(1,2))

hist(k)

scaleFreePlot(k,main="Check Scale free topology\n")

dev.off()

TOM = TOMsimilarity(adjacency)

dissTOM = 1-TOM

geneTree = hclust(as.dist(dissTOM), method = "average");

pdf(file="4_gene_clustering.pdf",width=12,height=9)

plot(geneTree, xlab="", sub="", main = "Gene clustering on TOM-based dissimilarity",

labels = FALSE, hang = 0.04)

dev.off()

minModuleSize = 60

dynamicMods = cutreeDynamic(dendro = geneTree, distM = dissTOM,

deepSplit = 2, pamRespectsDendro = FALSE,

minClusterSize = minModuleSize);

table(dynamicMods)

dynamicColors = labels2colors(dynamicMods)

table(dynamicColors)

pdf(file="5_Dynamic_Tree.pdf",width=8,height=6)

plotDendroAndColors(geneTree, dynamicColors, "Dynamic Tree Cut",

dendroLabels = FALSE, hang = 0.03,

addGuide = TRUE, guideHang = 0.05,

main = "Gene dendrogram and module colors")

dev.off()

MEList = moduleEigengenes(datExpr0, colors = dynamicColors)

MEs = MEList$eigengenes

MEDiss = 1-cor(MEs);

METree = hclust(as.dist(MEDiss), method = "average")

pdf(file="6_Clustering_module.pdf",width=7,height=6)

plot(METree, main = "Clustering of module eigengenes",

xlab = "", sub = "")

MEDissThres = 0.25

abline(h=MEDissThres, col = "red")

dev.off()

merge = mergeCloseModules(datExpr0, dynamicColors, cutHeight = MEDissThres, verbose = 3)

mergedColors = merge$colors

mergedMEs = merge$newMEs

pdf(file="7_merged_dynamic.pdf", width = 9, height = 6)

plotDendroAndColors(geneTree, mergedColors,"Dynamic Tree Cut",

dendroLabels = FALSE, hang = 0.03,

addGuide = TRUE, guideHang = 0.05,

main = "Gene dendrogram and module colors")

dev.off()

moduleColors = mergedColors

table(moduleColors)

colorOrder = c("grey", standardColors(50))

moduleLabels = match(moduleColors, colorOrder)-1

MEs = mergedMEs

nGenes = ncol(datExpr0)

nSamples = nrow(datExpr0)

moduleTraitCor = cor(MEs, datTraits, use = "p")

moduleTraitPvalue = corPvalueStudent(moduleTraitCor, nSamples)

pdf(file="8_Module_trait.pdf", width=6, height=5.5)

textMatrix = paste(signif(moduleTraitCor, 2), "\n(",

signif(moduleTraitPvalue, 1), ")", sep = "")

dim(textMatrix) = dim(moduleTraitCor)

par(mar = c(5, 10, 3, 3))

labeledHeatmap(Matrix = moduleTraitCor,

xLabels = names(datTraits),

yLabels = names(MEs),

ySymbols = names(MEs),

colorLabels = FALSE,

colors = blueWhiteRed(50),

textMatrix = textMatrix,

setStdMargins = FALSE,

cex.text = 0.5,

zlim = c(-1,1),

main = paste("Module-trait relationships"))

dev.off()

modNames = substring(names(MEs), 3)

geneModuleMembership = as.data.frame(cor(datExpr0, MEs, use = "p"))

MMPvalue = as.data.frame(corPvalueStudent(as.matrix(geneModuleMembership), nSamples))

names(geneModuleMembership) = paste("MM", modNames, sep="")

names(MMPvalue) = paste("p.MM", modNames, sep="")

traitNames=names(datTraits)

geneTraitSignificance = as.data.frame(cor(datExpr0, datTraits, use = "p"))

GSPvalue = as.data.frame(corPvalueStudent(as.matrix(geneTraitSignificance), nSamples))

names(geneTraitSignificance) = paste("GS.", traitNames, sep="")

names(GSPvalue) = paste("p.GS.", traitNames, sep="")

y=datTraits[,1]

GS1=as.numeric(cor(y, datExpr0, use="p"))

GeneSignificance=abs(GS1)

ModuleSignificance=tapply(GeneSignificance, mergedColors, mean, na.rm=T)

pdf(file="9_GeneSignificance.pdf", width=11, height=7)

plotModuleSignificance(GeneSignificance, mergedColors)

dev.off()

trait="Treat"

traitColumn=match(trait,traitNames)

for (module in modNames){

column = match(module, modNames)

moduleGenes = moduleColors==module

if (nrow(geneModuleMembership[moduleGenes,]) > 1){

outPdf=paste("10_", trait, "_", module,".pdf",sep="")

pdf(file=outPdf,width=7,height=7)

par(mfrow = c(1,1))

verboseScatterplot(abs(geneModuleMembership[moduleGenes, column]),

abs(geneTraitSignificance[moduleGenes, traitColumn]),

xlab = paste("Module Membership in", module, "module"),

ylab = paste("Gene significance for ",trait),

main = paste("Module membership vs. gene significance\n"),

cex.main = 1.2, cex.lab = 1.2, cex.axis = 1.2, col = module)

abline(v=0.8,h=0.5,col="red")

dev.off()

}

}

probes = colnames(datExpr0)

geneInfo0 = data.frame(probes= probes,

moduleColor = moduleColors)

for (Tra in 1:ncol(geneTraitSignificance))

{

oldNames = names(geneInfo0)

geneInfo0 = data.frame(geneInfo0, geneTraitSignificance[,Tra],

GSPvalue[, Tra])

names(geneInfo0) = c(oldNames,names(geneTraitSignificance)[Tra],

names(GSPvalue)[Tra])

}

for (mod in 1:ncol(geneModuleMembership))

{

oldNames = names(geneInfo0)

geneInfo0 = data.frame(geneInfo0, geneModuleMembership[,mod],

MMPvalue[, mod])

names(geneInfo0) = c(oldNames,names(geneModuleMembership)[mod],

names(MMPvalue)[mod])

}

geneOrder =order(geneInfo0$moduleColor)

geneInfo = geneInfo0[geneOrder, ]

write.table(geneInfo, file = "GS_MM.xls",sep="\t",row.names=F)

for (mod in 1:nrow(table(moduleColors)))

{

modules = names(table(moduleColors))[mod]

probes = colnames(datExpr0)

inModule = (moduleColors == modules)

modGenes = probes[inModule]

write.table(modGenes, file =paste0("module_",modules,".txt"),sep="\t",row.names=F,col.names=F,quote=F)

}

geneSigFilter=0.5

moduleSigFilter=0.8

datMM=cbind(geneModuleMembership, geneTraitSignificance)

datMM=datMM[abs(datMM[,ncol(datMM)])>geneSigFilter,]

for(mmi in colnames(datMM)[1:(ncol(datMM)-2)]){

dataMM2=datMM[abs(datMM[,mmi])>moduleSigFilter,]

write.table(row.names(dataMM2), file =paste0("hubGenes_",mmi,".txt"),sep="\t",row.names=F,col.names=F,quote=F)

}

**Figure3C**

library(venn)

diffFile="diff.txt"

wgcnaFile="hubGenes_MMpink.txt"

setwd("C:\\biowolf\\wgcnaDiagnostic\\12.venn")

geneList=list()

rt=read.table(diffFile, header=T, sep="\t", check.names=F)

geneNames=as.vector(rt[,1])

uniqGene=unique(geneNames)

geneList[["Difference"]]=uniqGene

rt=read.table(wgcnaFile, header=F, sep="\t", check.names=F)

geneNames=as.vector(rt[,1])

uniqGene=unique(geneNames)

geneList[["WGCNA"]]=uniqGene

mycol=c("blue2","red2")

pdf(file="venn.pdf", width=5, height=5)

venn(geneList,col=mycol[1:length(geneList)],zcolor=mycol[1:length(geneList)],box=F,ilabels=F)

dev.off()

intersectGenes=Reduce(intersect,geneList)

write.table(file="interGenes.txt", intersectGenes, sep="\t", quote=F, col.names=F, row.names=F)

**Figure3D-E**

set.seed(111)

library(glmnet)

expFile="normalize.txt"

geneFile="interGenes.txt"

setwd("C:\\biowolf\\wgcnaDiagnostic\\13.lasso")

rt=read.table(expFile, header=T, sep="\t", check.names=F, row.names=1)

geneRT=read.table(geneFile, header=F, sep="\t", check.names=F)

rt=rt[as.vector(geneRT[,1]),]

rt=t(rt)

x=as.matrix(rt)

y=gsub("(.*)\\_(.*)", "\\2", row.names(rt))

fit=glmnet(x, y, family = "binomial", alpha=1)

pdf(file="lasso.pdf", width=6, height=5.5)

plot(fit)

dev.off()

cvfit=cv.glmnet(x, y, family="binomial", alpha=1,type.measure='deviance',nfolds = 10)

pdf(file="cvfit.pdf", width=6, height=5.5)

plot(cvfit)

dev.off()

coef=coef(fit, s = cvfit$lambda.min)

index=which(coef != 0)

lassoGene=row.names(coef)[index]

lassoGene=lassoGene[-1]

write.table(lassoGene, file="LASSO.gene.txt", sep="\t", quote=F, row.names=F, col.names=F)

**Figure4A-C**

library(limma)

library(ggpubr)

expFile="normalize.txt"

geneFile="LASSO.gene.txt"

setwd("C:\\biowolf\\wgcnaDiagnostic\\14.boxplot")

rt=read.table(expFile, header=T, sep="\t", check.names=F)

rt=as.matrix(rt)

rownames(rt)=rt[,1]

exp=rt[,2:ncol(rt)]

dimnames=list(rownames(exp), colnames(exp))

data=matrix(as.numeric(as.matrix(exp)), nrow=nrow(exp), dimnames=dimnames)

data=avereps(data)

geneRT=read.table(geneFile, header=F, sep="\t", check.names=F)

data=data[as.vector(geneRT[,1]),,drop=F]

Type=gsub("(.*)\\_(.*)", "\\2", colnames(data))

Type=ifelse(Type=="con", "Con", "Treat")

my_comparisons=list()

my_comparisons[[1]]=levels(factor(Type))

for(i in row.names(data)){

#data[i,][data[i,]>quantile(data[i,], 0.99)]=quantile(data[i,], 0.99)

rt1=data.frame(expression=data[i,], Type=Type)

boxplot=ggboxplot(rt1, x="Type", y="expression", color="Type",

xlab="",

ylab=paste(i, "expression"),

legend.title="",

palette = c("blue", "red"),

add = "jitter")+

stat_compare_means(comparisons = my_comparisons,symnum.args=list(cutpoints=c(0, 0.001, 0.01, 0.05, 1), symbols=c("***", "**", "*", "ns")), label="p.signif")

#stat_compare_means(comparisons = my_comparisons)

pdf(file=paste0("boxplot.",i,".pdf"), width=5, height=4.5)

print(boxplot)

dev.off()

}

**Figure4D-F**

library(limma)

library(ggpubr)

expFile="GSE18179.txt"

conFile="GSE18179_s1.txt"

treatFile="GSE18179_s2.txt"

geneFile="LASSO.gene.txt"

setwd("C:\\Users\\lenovo\\Desktop\\146wgcna\\15.testDiff")

rt=read.table(expFile, header=T, sep="\t", check.names=F)

rt=as.matrix(rt)

rownames(rt)=rt[,1]

exp=rt[,2:ncol(rt)]

dimnames=list(rownames(exp), colnames(exp))

data=matrix(as.numeric(as.matrix(exp)), nrow=nrow(exp), dimnames=dimnames)

rt=avereps(data)

qx=as.numeric(quantile(rt, c(0, 0.25, 0.5, 0.75, 0.99, 1.0), na.rm=T))

LogC=( (qx[5]>100) || ( (qx[6]-qx[1])>50 && qx[2]>0) )

if(LogC){

rt[rt<0]=0

rt=log2(rt+1)}

data=normalizeBetweenArrays(rt)

con=read.table(conFile, header=F, sep="\t", check.names=F)

treat=read.table(treatFile, header=F, sep="\t", check.names=F)

conData=data[,as.vector(con[,1])]

treatData=data[,as.vector(treat[,1])]

data=cbind(conData, treatData)

conNum=ncol(conData)

treatNum=ncol(treatData)

Type=c(rep("con",conNum), rep("treat",treatNum))

outData=rbind(id=paste0(colnames(data),"_",Type),data)

write.table(outData, file="test.normalize.txt", sep="\t", quote=F, col.names=F)

geneRT=read.table(geneFile, header=F, sep="\t", check.names=F)

data=data[as.vector(geneRT[,1]),,drop=F]

Type=c(rep("Con",conNum), rep("Treat",treatNum))

my_comparisons=list()

my_comparisons[[1]]=levels(factor(Type))

for(i in row.names(data)){

#data[i,][data[i,]>quantile(data[i,], 0.99)]=quantile(data[i,], 0.99)

rt1=data.frame(expression=data[i,], Type=Type)

boxplot=ggboxplot(rt1, x="Type", y="expression", color="Type",

xlab="",

ylab=paste(i, "expression"),

legend.title="",

palette = c("blue", "red"),

add = "jitter")+

stat_compare_means(comparisons = my_comparisons,symnum.args=list(cutpoints=c(0, 0.001, 0.01, 0.05, 1), symbols=c("***", "**", "*", "ns")), label="p.signif")

#stat_compare_means(comparisons = my_comparisons)

pdf(file=paste0("boxplot.",i,".pdf"), width=5, height=4.5)

print(boxplot)

dev.off()

}

**Figure5A-C**

library(pROC)

expFile="normalize.txt"

geneFile="LASSO.gene.txt"

setwd("C:\\biowolf\\wgcnaDiagnostic\\16.ROC")

rt=read.table(expFile, header=T, sep="\t", check.names=F, row.names=1)

y=gsub("(.*)\\_(.*)", "\\2", colnames(rt))

y=ifelse(y=="con", 0, 1)

geneRT=read.table(geneFile, header=F, sep="\t", check.names=F)

for(x in as.vector(geneRT[,1])){

roc1=roc(y, as.numeric(rt[x,]))

ci1=ci.auc(roc1, method="bootstrap")

ciVec=as.numeric(ci1)

pdf(file=paste0("ROC.",x,".pdf"), width=5, height=5)

plot(roc1, print.auc=TRUE, col="red", legacy.axes=T, main=x)

text(0.39, 0.43, paste0("95% CI: ",sprintf("%.03f",ciVec[1]),"-",sprintf("%.03f",ciVec[3])), col="red")

dev.off()

}

**Figure5D-F**

library(pROC)

expFile="test.normalize.txt"

geneFile="LASSO.gene.txt"

setwd("C:\\biowolf\\wgcnaDiagnostic\\17.testROC")

rt=read.table(expFile, header=T, sep="\t", check.names=F, row.names=1)

y=gsub("(.*)\\_(.*)", "\\2", colnames(rt))

y=ifelse(y=="con", 0, 1)

geneRT=read.table(geneFile, header=F, sep="\t", check.names=F)

for(x in as.vector(geneRT[,1])){

roc1=roc(y, as.numeric(rt[x,]))

ci1=ci.auc(roc1, method="bootstrap")

ciVec=as.numeric(ci1)

pdf(file=paste0("ROC.",x,".pdf"), width=5, height=5)

plot(roc1, print.auc=TRUE, col="red", legacy.axes=T, main=x)

text(0.39, 0.43, paste0("95% CI: ",sprintf("%.03f",ciVec[1]),"-",sprintf("%.03f",ciVec[3])), col="red")

dev.off()

}

**Figure6A**

library(pheatmap)

inputFile="ssGSEA.result.txt"

setwd("C:\\biowolf\\wgcnaDiagnostic\\19.heatmap")

rt=read.table(inputFile, header=T, sep="\t", check.names=F, row.names=1)

con=grepl("_con", colnames(rt), ignore.case=T)

treat=grepl("_treat", colnames(rt), ignore.case=T)

conData=rt[,con]

treatData=rt[,treat]

conNum=ncol(conData)

treatNum=ncol(treatData)

data=cbind(conData,treatData)

Type=c(rep("Con",conNum), rep("Treat",treatNum))

names(Type)=colnames(data)

Type=as.data.frame(Type)

pdf(file="heatmap.pdf", width=8, height=5)

pheatmap(data,

annotation=Type,

color=colorRampPalette(c(rep("blue",3), "white", rep("red",3)))(50),

cluster_cols=F,

show_colnames=F,

scale="row",

fontsize = 6,

fontsize_row=6,

fontsize_col=6)

dev.off()

**Figure6B**

library(vioplot)

inputFile="ssGSEA.result.txt"

setwd("C:\\biowolf\\wgcnaDiagnostic\\20.vioplot")

rt=read.table(inputFile, header=T, sep="\t", check.names=F, row.names=1)

con=grepl("_con", colnames(rt), ignore.case=T)

treat=grepl("_treat", colnames(rt), ignore.case=T)

conData=rt[,con]

treatData=rt[,treat]

conNum=ncol(conData)

treatNum=ncol(treatData)

rt=t(cbind(conData,treatData))

outTab=data.frame()

pdf(file="vioplot.pdf", width=13, height=8.5)

par(las=1,mar=c(10,6,3,3))

x=c(1:ncol(rt))

y=c(1:ncol(rt))

plot(x,y,

xlim=c(0,(3*ncol(rt)-3)),ylim=c(min(rt),max(rt)+0.05),

main="",xlab="", ylab="Fraction",

pch=21,

col="white",

xaxt="n")

for(i in 1:ncol(rt)){

if(sd(rt[1:conNum,i])==0){

rt[1,i]=0.00001

}

if(sd(rt[(conNum+1):(conNum+treatNum),i])==0){

rt[(conNum+1),i]=0.00001

}

conData=rt[1:conNum,i]

treatData=rt[(conNum+1):(conNum+treatNum),i]

vioplot(conData,at=3*(i-1),lty=1,add = T,col = 'blue')

vioplot(treatData,at=3*(i-1)+1,lty=1,add = T,col = 'red')

wilcoxTest=wilcox.test(conData,treatData)

p=wilcoxTest$p.value

if(p<0.05){

cellPvalue=cbind(Cell=colnames(rt)[i],pvalue=p)

outTab=rbind(outTab,cellPvalue)

}

mx=max(c(conData,treatData))

lines(c(x=3*(i-1)+0.2,x=3*(i-1)+0.8),c(mx,mx))

text(x=3*(i-1)+0.5, y=mx+0.02, labels=ifelse(p<0.001, paste0("p<0.001"), paste0("p=",sprintf("%.03f",p))), cex = 0.8)

}

legend("topright",

c("Con", "Treat"),

lwd=3,bty="n",cex=1,

col=c("blue","red"))

text(seq(1,(3*ncol(rt)-2),3),-0.075,xpd = NA,labels=colnames(rt),cex = 1,srt = 45,pos=2)

dev.off()

write.table(outTab,file="immuneDiff.xls",sep="\t",row.names=F,quote=F)

**Figure6C**

library(limma)

library(reshape2)

library(tidyverse)

library(ggplot2)

expFile="normalize.txt"

geneFile="LASSO.gene.txt"

immFile="ssGSEA.result.txt"

setwd("C:\\biowolf\\wgcnaDiagnostic\\21.immuneCor")

immune=read.table(immFile, header=T, sep="\t", check.names=F, row.names=1)

immune=t(immune)

rt=read.table(expFile, header=T, sep="\t", check.names=F, row.names=1)

geneRT=read.table(geneFile, header=F, sep="\t", check.names=F)

exp=t(rt[as.vector(geneRT[,1]),])

sameSample=intersect(row.names(immune), row.names(exp))

treat=grepl("_treat", sameSample, ignore.case=T)

sameSample=sameSample[treat]

immune=immune[sameSample,,drop=F]

exp=exp[sameSample,,drop=F]

outTab=data.frame()

for(cell in colnames(immune)){

for(gene in colnames(exp)){

x=as.numeric(immune[,cell])

y=as.numeric(exp[,gene])

corT=cor.test(x,y,method="spearman")

cor=corT$estimate

pvalue=corT$p.value

text=ifelse(pvalue<0.001,"***",ifelse(pvalue<0.01,"**",ifelse(pvalue<0.05,"*","")))

outTab=rbind(outTab,cbind(Gene=gene, Immune=cell, cor, text, pvalue))

}

}

outTab$cor=as.numeric(outTab$cor)

pdf(file="cor.pdf", width=7, height=6)

ggplot(outTab, aes(Gene, Immune)) +

geom_tile(aes(fill = cor), colour = "grey", size = 1)+

scale_fill_gradient2(low = "#5C5DAF", mid = "white", high = "#EA2E2D") +

geom_text(aes(label=text),col ="black",size = 3) +

theme_minimal() +

theme(axis.title.x=element_blank(), axis.ticks.x=element_blank(), axis.title.y=element_blank(),

axis.text.x = element_text(angle = 45, hjust = 1, size = 10, face = "bold"),体

axis.text.y = element_text(size = 10, face = "bold")) +

labs(fill =paste0("*** p<0.001","\n", "** p<0.01","\n", " * p<0.05","\n", "\n","Correlation")) +

scale_x_discrete(position = "bottom")

dev.off()
